# Projects to support people with dementia, Shanghai, China

**DOI:** 10.2471/BLT.25.294416

**Published:** 2025-12-02

**Authors:** Qiuling An, Fei Sun

**Affiliations:** a China Center for Modern City Studies, School of Social Development, East China Normal University, Shanghai, China.; b School of Social Work, Michigan State University, 655 Auditorium Rd, East Lansing, MI 48824, United States of America.

## Abstract

**Problem:**

China is experiencing rapid population ageing, placing increasing pressure on health-care systems, social services and families.

**Approach:**

In 2019, the Shanghai Civil Affairs Bureau launched a citywide initiative to create dementia-friendly communities that integrate nonpharmacological interventions as an essential component. The bureau invited local agencies to apply for funding and design locally tailored interventions for people aged 55 years or older with cognitive impairment. Approved projects received funding over a three-year period. The bureau conducted annual assessments of the projects, and projects rated as excellent were showcased during public awareness campaigns for dementia.

**Local setting:**

Shanghai, one of China' s most populous and rapidly ageing cities, has an estimated 300 000 people with dementia.

**Relevant changes:**

By 2023, all 216 subdistricts and townships in Shanghai had implemented a project. These projects provided cognitive training and education and incorporated one or more activities for participants, such as horticulture, games, music, exercise and aromatherapy. Among the surveyed participants in 44 projects conducted in 2023 and 2024, 85.1% (275/323) reported being satisfied or very satisfied, and 90.1% (291/323) believed the interventions met their needs.

**Lessons learnt:**

Shanghai’s publicly funded model enabled citywide dementia-friendly projects, which were highly appreciated by participants. Future efforts should strengthen academic collaboration for evidence-based practice, and adopt standardized protocols with validated measures to ensure consistent assessment and readiness for scale-up. To reinforce progress, the bureau launched the 2025–2030 implementation plan to sustain continued funding and provide technical support.

## Résumé

**Problème:**

La Chine connaît un vieillissement rapide de sa population, ce qui exerce une pression croissante sur les systèmes de santé, les services sociaux et les familles.

**Approche:**

En 2019, le Bureau des affaires civiles de Shanghai a lancé une initiative à l’échelle de la ville visant à créer des communautés adaptées aux personnes atteintes de démence et qui intègrent des interventions non pharmacologiques comme élément central. Ce bureau a invité les agences locales à demander des financements et à concevoir des interventions adaptées aux personnes âgées de 55 ans et plus souffrant de troubles cognitifs. Les projets approuvés ont reçu un financement sur une période de trois ans. Le bureau a procédé à des évaluations annuelles des projets, et ceux jugés excellents ont été présentés lors de campagnes de sensibilisation à la démence.

**Environnement local:**

Shanghai, l’une des villes les plus peuplées de Chine mais où la population vieillit le plus rapidement, compte environ 300 000 personnes atteintes de démence.

**Changements significatifs:**

En 2023, les 216 sous-districts et communes de Shanghai ont chacun participé à un projet parmi un panel de différents projets. Ceux-ci prévoyaient une formation et une éducation cognitives et intégraient une ou plusieurs activités pour les participants, telles que l’horticulture, les jeux, la musique, l’exercice physique et l’aromathérapie. Parmi les participants interrogés dans le cadre de 44 projets menés en 2023 et 2024, 85,1% (275/323) se sont déclarés satisfaits ou très satisfaits, et 90,1% (291/323) ont estimé que les interventions répondaient à leurs besoins.

**Leçons tirées:**

Le modèle shanghaïen financé par des fonds publics a permis de mettre en place, à l’échelle de la ville, des projets adaptés aux personnes atteintes de démence, qui ont été très appréciés par les participants. Les efforts futurs devraient renforcer la collaboration universitaire pour une pratique fondée sur des données scientifiques et adopter des protocoles standardisés avec des mesures validées afin de garantir une évaluation cohérente et une préparation à la mise à l’échelle. Afin de consolider les avancées, le bureau a lancé le plan de mise en œuvre 2025–2030 afin de maintenir un financement continu et de fournir un soutien technique.

## Resumen

**Situación:**

China está experimentando un rápido envejecimiento de la población, lo que ejerce una creciente presión sobre los sistemas de atención de salud, los servicios sociales y las familias.

**Enfoque:**

En 2019, la Oficina de Asuntos Civiles de Shanghái puso en marcha una iniciativa municipal para crear comunidades amigables con las personas con demencia que integraran intervenciones no farmacológicas como componente esencial. La oficina invitó a las agencias locales a solicitar financiación y diseñar intervenciones adaptadas localmente para personas de ≥55 años con deterioro cognitivo. Los proyectos aprobados recibieron financiación durante un periodo de tres años. La oficina realizó evaluaciones anuales de los proyectos y aquellos calificados como excelentes se presentaron durante campañas de sensibilización pública sobre la demencia.

**Marco regional:**

Shanghái, una de las ciudades más pobladas y de envejecimiento más rápido de China, cuenta con aproximadamente 300 000 personas con demencia.

**Cambios importantes:**

Para 2023, los 216 subdistritos y municipios de Shanghái habían implementado un proyecto. Estos proyectos ofrecieron entrenamiento y educación cognitiva e incorporaron una o más actividades para los participantes, como horticultura, juegos, música, ejercicio y aromaterapia. Entre los participantes encuestados en 44 proyectos realizados en 2023 y 2024, el 85,1% (275/323) declaró estar satisfecho o muy satisfecho y el 90,1% (291/323) consideró que las intervenciones respondían a sus necesidades.

**Lecciones aprendidas:**

El modelo financiado con fondos públicos de Shanghái permitió la implementación de proyectos municipales amigables con las personas con demencia, que fueron muy valorados por los participantes. Los esfuerzos futuros deberían fortalecer la colaboración académica para promover prácticas basadas en la evidencia y adoptar protocolos estandarizados con medidas validadas que garanticen evaluaciones consistentes y preparación para la ampliación. Para consolidar los avances, la oficina lanzó el plan de implementación 2025-2030, destinado a mantener la financiación continua y proporcionar apoyo técnico.

## ملخص

**المشكلة:**

تمر الصين بتقدم سريع في السن للسكان، مما يُشكل ضغطًا متزايدًا على أنظمة الرعاية الصحية، والخدمات الاجتماعية، والأسر.

**الأسلوب:**

في عام 2019، أطلق مكتب الشؤون المدنية في شنغهاي مبادرة على مستوى المدينة لإنشاء مجتمعات صديقة للمصابين بالخرف، تقوم بدمج التدخلات غير الدوائية كعنصر أساسي. دعا المكتب الوكالات المحلية لتقديم طلبات التمويل وتطوير تدخلات مُصممة خصيصًا محليًا للأشخاص الذين تبلغ أعمارهم 55 عامًا ويعانون من ضعف الإدراك. حصلت المشروعات المُعتمدة على تمويل على مدى ثلاث سنوات. وأجرى المكتب تقييمات سنوية للمشروعات، وعُرضت المشاريع المُصنفة على أنها ممتازة خلال حملات التوعية العامة بالخرف.

**المواقع المحلية:**

تُقدر أعداد المصابين بالخرف في شنغهاي، إحدى أكثر مدن الصين اكتظاظًا بالسكان وتسارعًا في معدلات الشيخوخة، بنحو 300 ألف شخص.

**التغيّرات ذات الصلة:**

بحلول عام 2023، كانت جميع المناطق الفرعية والبلدات الـ 216 في شنغهاي قد نفذت مشروعًا. قدمت هذه المشروعات تدريبًا وتعليمًا معرفيًا، وتضمنت نشاطًا واحدًا أو أكثر للمشاركين، مثل البستنة والألعاب والموسيقى والتمارين الرياضية والعلاج بالروائح. من بين المشاركين الذين شملهم الاستطلاع في 44 مشروعًا نُفذت في عامي 2023 و2024، أفاد %85.1 (275/323) بأنهم راضون أو راضون للغاية، بينما رأى %90.1 (291/323) أن التدخلات تلبي احتياجاتهم.

**الدروس المستفادة:**

إن النموذج الممول من القطاع العام في شنغهاي أدى للتمكين من تنفيذ مشاريع على مستوى المدينة، بحيث تُراعي مرضى الخرف، وقد لاقت استحسانًا كبيرًا من المشاركين. يجب أن تُعزز الجهود المستقبلية التعاون الأكاديمي من أجل ممارسات قائمة على الأدلة، وأن تتبنى بروتوكولات قياسية مع تدابير مُعتمدة لضمان التقييم المتسق والاستعداد للتوسع. ولتعزيز التقدم، أطلق المكتب خطة التنفيذ 2025-2030 لدعم استمرار التمويل وتقديم الدعم الفني.

## 摘要

**问题:**

中国目前正处于快速人口老龄化阶段，这导致卫生保健系统、社会服务机构和各个家庭所面临压力与日俱增。

**方法:**

上海市民政局于 2019 年在全市范围内推行了一项倡议，旨在创建强调非药物干预的痴呆症患者关爱社区。上海市民政局邀请了各地方机构以申请资金，并针对年满 55 岁且存在认知功能损害的老年人制定符合当地实际情况的干预措施。已获批项目获得了为期三年的资金支持。上海市民政局对所有支持项目进行了年度评估，并在痴呆症相关公众意识宣传活动中展示了被评定为优秀的项目。

**当地状况:**

上海是中国人口最多且正处于快速老龄化阶段的城市之一，据估计，全市共有 300,000 名痴呆症患者。

**相关变化:**

截至 2023 年，上海市的 216 个街道和乡镇均已推行支持项目。通过这些项目，参与者可接受融入了一种或多种活动元素的认知训练和教育，例如园艺、游戏、音乐、运动和芳香疗法。针对 2023 年和 2024 年开展的 44 个项目所涉及参与者开展的调查结果显示，85.1% (275/323) 的参与者报告称其满意或非常满意，90.1% (291/323) 的参与者认为干预措施符合其需求。

**经验教训:**

中国上海这种以政府资助模式在全市范围内开展的痴呆症患者关爱项目得到了参与者的高度赞赏。未来应着眼于加强循证实践相关学术合作，并采取包含已验证措施的标准化协议，以确保评估的一致性并为扩大规模做好相应准备。为了推动项目进度，上海市民政局推行了 2025-2030 年实施计划，旨在确保持续获得资金支持和提供技术支持服务。

## Резюме

**Проблема:**

Китай сталкивается с быстрым старением населения, что создает растущую нагрузку на системы здравоохранения, социальные службы и семьи.

**Подход:**

В 2019 году Шанхайское бюро по гражданским делам инициировало общегородскую программу по созданию общин, в которых положительно относились бы к людям с деменцией. Ключевым компонентом этой инициативы стало внедрение нефармакологических вмешательств. Бюро предложило местным агентствам подавать заявки на гранты для разработки мероприятий, направленных на поддержку лиц в возрасте от 55 лет с когнитивными нарушениями, с учетом местной специфики. Утвержденные проекты получали финансирование на протяжении трех лет. Бюро проводило ежегодное оценивание проектов, и те из них, которые получили отличные оценки, демонстрировались в ходе публичных информационных кампаний по вопросам деменции.

**Местные условия:**

В Шанхае, одном из наиболее густонаселенных и быстро стареющих городов Китая, по оценкам проживает около 300 000 людей с деменцией.

**Осуществленные перемены:**

По состоянию на 2023 год все 216 районов и поселений Шанхая присоединились к рассматриваемому проекту, в рамках которого предусматривались когнитивные тренинги и обучение. Участникам также предлагали одно или несколько занятий, таких как садоводство, игры, музыка, физические упражнения и ароматерапия. Среди опрошенных участников 44 проектов, реализованных в 2023 и 2024 годах, 85,1% (275/323) были удовлетворены или весьма удовлетворены результатами, и 90,1% (291/323) считали, что проведенные мероприятия соответствуют их потребностям.

**Выводы:**

Шанхайская модель с общественным финансированием позволила создать в масштабах всего города проекты, основанные на принципах доброжелательного отношения к людям с деменцией, и эти проекты были высоко оценены участниками. В дальнейшем следует сосредоточить усилия на укреплении академического сотрудничества с целью выработки практики, опирающейся на доказательную базу, и принять стандартизованные протоколы с валидированными измеряемыми параметрами, что позволило бы проводить единообразную оценку и обеспечило бы потенциальную масштабируемость. Для закрепления достигнутого прогресса бюро запустило план действий на 2025–2030 годы, направленный на обеспечение устойчивого финансирования и технической поддержки проекта.

## Introduction

Supporting people living with dementia is a global public health priority.^1^ In 2020, an estimated 55 million people lived with dementia worldwide, a figure projected to reach 153 million by 2050.^2^^,^^3^

In China, the number of people living with dementia is projected to rise from 17 million in 2025 to 66 million in 2050, which will account for one tenth of the older population in the country.^4^ However, diagnosis rates remain low: only an estimated 14.0% of people living with mild dementia receive a diagnosis; 34.0% of those with severe dementia are diagnosed; and 49.0% of diagnoses are misattributed to normal ageing, leading to late-stage diagnosis.^5^ Limited public awareness of dementia, constrained access to formal services and a culture that emphasizes family-based care make family members the main caregivers. 

To improve dementia care, Putuo District in Shanghai piloted a national dementia-friendly community project in 2015. Based on this pilot project, the Shanghai municipality launched a citywide initiative in 2019, which is aligned with the Chinese national dementia plan.^6^ A key component of this plan is to enhance community-based support for people with dementia and their caregivers, which aligns with action goals 2 and 5 of the World Health Organization’s (WHO) *Global action plan on the public health response to dementia 2017–2025*.^1^ Action 2 emphasizes increasing dementia awareness and community friendliness, while action 5 stresses caregiver support. 

Here, we report on Shanghai’s dementia-friendly community projects, based on agency data, existing evaluation documents and first-hand surveys of participants.

## Local setting

Shanghai is China’s most populous city and its population of 25 million has the country’s oldest age structure. In 2023, 29.4% (7 313 100/24 874 500) of its residents were 65 years or older, with an estimated 300 000 people living with Alzheimer disease or related dementias.^7^ However, one study found only 6.2% (59/951) of adults aged 55 years or older sought medical care for memory concerns.^8^

Shanghai has 16 districts and 216 subdistricts or towns under a three-tier city–district–subdistrict governance system. The Shanghai Municipal Bureau of Civil Affairs, through its Division of Ageing Services, regulates and supervises institutional, community and home-based elder-care services.

## Approaches

In 2019, the Municipal Bureau of Civil Affairs introduced the dementia-friendly initiative, a community-driven, psychosocial and participatory model that promotes social connection, cognitive stimulation and caregiver inclusion. The initiative prioritizes nonpharmacological interventions to enhance quality of life for people living with dementia.^9^^,^^10^

To initiate a dementia-friendly initiative, subdistrict, village and town governments invited local social service and health-care agencies, including elder-care facilities, to apply for funds to run the projects. The application needed to include a project description with a target population, proposed approaches and the intended outputs and outcomes. The agencies were responsible for designing the projects using the bureau’s guidelines and drew on proven interventions for dementia prevention, such as cognitive training, physical activity, music and reminiscence, nutrition support and psychosocial interventions.^11^^,^^12^ The projects typically include dementia screening, public awareness and social support. When agencies received funding, they conducted local needs assessments of older adults affected by cognitive impairment, their family caregivers and agency staff capacity to inform project content and delivery. Some agencies sought expert consultation, such as collaborating with a university, as needed. Most projects are staffed by three to five people, including a social worker or counsellor, a site coordinator and volunteer(s). 

Adults aged 55 years or older who are living with dementia or mild cognitive impairment, and reside either in the community or in residential care facilities, can participate. 

To inform eligible adults about the project, the agencies posted notices on bulletin boards at community activity centres. Many agencies also shared information through their official WeChat (Tencent Holdings Ltd, Shenzhen, China) public accounts, while word of mouth was a common way to spread the information.

Each project usually runs 12-week programmes, three to four times per year. Participants meet once a week for about an hour in a group comprising about 50 participants.

Each approved project received city government funding for three years, with a requirement of the same amount matched from the district government. Funds may be used for agency infrastructure upgrades, additional staffing and subsidies for volunteers that facilitate programming operation. Awards are disbursed annually, contingent on passing an annual evaluation set by the bureau. This evaluation combines a self-assessment with a supervisory review. Each year, agencies submit their self-assessments of funded projects to subdistricts, village or town governments, which forward them to the bureau. The bureau also convenes an evaluation team, consisting of administrative leaders, university experts, senior managers and geriatric physicians, to conduct the supervisory review, which includes site visits, hearing agency reports, observing activities and rating projects based on team capacity, sustainability of resource development and distinctive project branding. Additionally, there is a mid-point evaluation conducted after the project has been running for one and a half years. This evaluation focuses on the impact achieved, using the same four rating categories as the annual review: excellent, good, meets expectations and does not meet expectations. Those projects rated as meeting the expectations or higher are designated as official dementia-friendly communities and remain eligible for ongoing support for the remaining year. If a project did not meet expectations, the agency had to submit a rectification plan to address the issues. The bureau considered projects rated as excellent as outstanding case examples and these projects were showcased during public awareness campaigns. 

Funded agencies receive 300 000 Chinese Yuan (about 45 000 United States dollars, US$) in the first year, and 150 000 Chinese Yuan (about US$ 22 500) in the second year to conduct their proposed activities to make their community dementia friendly. Funding for the third year depends on the results of the mid-term evaluation. The cost of running the nonpharmaceutical interventions is approximately 100 000 Chinese Yuan (about US$ 15 000) per year.

To report on these projects, we obtained secondary data from the bureau and agencies. We also engaged with agencies that had operated nonpharmacological intervention projects for at least one year, and were willing to provide additional information as well as to help administer an in-person paper-based survey free-of-charge to 323 previous participants from 44 projects. The survey was conducted between March and June 2025 and survey participants provided feedback on the nonpharmacological interventions, including ratings of satisfaction, perceived usefulness and additional comments. All procedures were approved by the Institutional Review Board of East China Normal University (IRB #20230427–1006).

## Relevant changes

The dementia-friendly initiative in Shanghai began with 28 project sites in 2019 and expanded to 216 projects by 2023, covering all subdistricts and townships in Shanghai. 

Box 1 presents an overview of 44 nonpharmacological intervention projects implemented in 2023 and 2024, targeting older adults with cognitive impairment or dementia. These projects were primarily psychosocial interventions and incorporated a range of integrated activities, including eight horticultural, seven game-based, six musical, five exercise and four aromatherapy activities. Five projects combined more than two activities. The remaining nine projects included other types of interventions, such as dietary and craft-based activities. The projects were implemented across diverse settings, including seven community service agencies, six social welfare elder-care institutions, five nongovernmental organizations and one hospital.

Box 1 **Overview of the 44 dementia-friendly projects in Shanghai, China, 2023–2024**

**Type of activities**

- Horticulture (eight projects): activities and discussions based on the Chinese philosophy of harmony between humans and nature.
- Games (seven projects): traditional cultural games linked to seasonal festivals, such as pitch-pot.
- Music (six projects): learning, singing and performing songs familiar to participants.
- Exercise (five projects): traditional Chinese exercises, for example Tai Chi, combined with thematic learning.
- Aromatherapy (four projects): learning, producing and using plant-based essential oils.
- Integrated (five projects): combining more than two activities.
- Other (nine projects): themes not included above, such as nutrition and handicrafts.

**Project design collaboration**

Most communities or agencies designed the project alone. However, five (11.4%) projects were co-designed by universities, agencies and communities.

**Intervention manual**

Most projects (39; 88.7%) developed an intervention manual before implementation.

**Participant recruitment**

Participants joined the project voluntarily following informal invitation shared on community bulletin boards or social media. Most projects (41; 93.2%) recruited participants either from the community where the agency was located or from nearby communities.

**Project sites**

Half of the projects were implemented in residential care facilities and the remainder in community activity centres. Forty (90.9%) of venues were within a 10-minute walk for participants.

**Project management**

All projects kept attendance records and post-session feedback logs. For group intervention facilitators, 30 (68.2%) projects provided supervision during implementation as well as process training.

Outcome evaluation

• Satisfaction surveys: 18 projects (40.9%)

• Pre- and post-assessments: 21 projects (47.8%)

• Follow-up assessments: five projects (11.4%)

• Quantitative analysis: four projects (9.1%)

Note: We obtained information about the projects from the Municipal Bureau of Civil Affairs and local agencies.

Across the 44 projects, participant responses were largely positive. Among 323 participants who provided feedback, 85.1% (275) individuals reported being satisfied or very satisfied with the project, and 90.1% (291) believed that the interventions met their needs. Overall, 85.4% (276) indicated that they had gained meaningful benefits and 69.7% (225) expressed a strong willingness to continue participating in these projects.

## Lessons learnt

Before municipal involvement, community projects for people with dementia were sporadic and largely initiated by local nongovernmental social service agencies and hospitals in Shanghai. Following increased awareness among the public, bolstered by public campaigns such as Alzheimer month, and the publication of WHO’s *Global action plan on the public health response to dementia 2017–2025*,^1^ the Shanghai municipal government recognized the challenges of dementia care and created the dementia-friendly initiative. In addition to providing initial funding, the municipal government requested local matched funds, tied continued funding to structured evaluations and publicly recognized exemplary projects. This combination of financing, accountability and recognition motivated subdistricts and townships to expand investment and deepen engagement in nonpharmacological interventions.

Projects that integrated person-centred, culturally familiar elements saw higher participant engagement, satisfaction and perceived usefulness. Older participants especially appreciated activities anchored in 1950s and 1960s music, traditional crafts and local daily routines, like sharing foods aligned with the 24 Chinese solar terms. These findings are consistent with evidence that empowerment-oriented, socially supportive interventions enhance engagement and psychosocial well-being for people living with dementia.^13^^,^^14^

Many community agencies found it challenging to provide effective interventions, and some sought collaboration with academic institutions for advice on designing, delivering and evaluating multidimensional interventions. Academic teams contributed to conceptual frameworks, structured manuals, staff training and monitoring the adherence to the implementation protocol; while local agencies ensured cultural fit, logistics and sustained delivery. The academic teams ensured that the project designs were based on the latest evidence for improving quality of life for older people with cognitive impairment. This collaboration also provided agencies with practical toolkits and trained staff that supported scale-up and sustainability of the projects.

Evaluation revealed variable implementation quality. Most projects lacked an explicit theory of change, standardized protocols and/or quality checks, and project outcome assessments were often restricted to participant attendance or satisfaction (Box 1). These shortcomings reflect agencies’ limited prior experience with intervention delivery and rigorous evaluation. While some agencies sought academic partners, many lacked the resources necessary to establish connections with a university or research institution. Recognizing these gaps, the municipal government’s recent 2025–2030 plan aims to establish a three-tier support system, involving municipal guidance, district capacity-building and subdistrict implementation for ongoing quality improvement.

Shanghai’s publicly funded model catalysed community action, leading to municipality-wide implementation of nonpharmacological, dementia-friendly projects that were highly appreciated by participants. Lessons learnt indicate that such interventions should include generation-relevant and culturally appropriate elements. For future projects, collaboration with academic institutions should be strengthened to support evidence-based practice, and standardized protocols with validated measures should be used to ensure consistent assessment and readiness for scale-up (Box 2). Taken together, Shanghai’s experience offers practical guidance for other municipalities seeking to advance dementia-friendly, community-based care.

Box 2 **Summary of main lessons learnt**

- To enhance acceptability and impact of the projects among persons living with dementia, community-based nonpharmacological interventions should incorporate generation-relevant and culturally appropriate elements.
- To promote evidence-based practice and support high-quality implementation across all projects, collaborations with academic institutions should be strengthened.
- Standardized protocols and validated measures are needed for consistent assessment frameworks to improve fidelity, comparability and readiness for replication and scale-up.
